# Application of Convolutional Neural Networks in an Automatic Judgment System for Tooth Impaction Based on Dental Panoramic Radiography

**DOI:** 10.3390/diagnostics15111363

**Published:** 2025-05-28

**Authors:** Ya-Yun Huang, Yi-Cheng Mao, Tsung-Yi Chen, Chiung-An Chen, Shih-Lun Chen, Yu-Jui Huang, Chun-Han Chen, Jun-Kai Chen, Wei-Chen Tu, Patricia Angela R. Abu

**Affiliations:** 1 Program on Semiconductor Manufacturing Technology Academy of Innovative Semiconductor and Sustainable Manufacturing, National Cheng Kung University, Tainan City 70101, Taiwan; m28124023@gs.ncku.edu.tw (Y.-Y.H.); wctu@gs.ncku.edu.tw (W.-C.T.); 2 Department of General Dentistry, Chang Gung Memorial Hospital, Taoyuan City 33305, Taiwan; louiszzzzz@cgmh.org.tw; 3 Department of Electronic Engineering, Feng Chia University, Taichung City 40724, Taiwan; 4 Department of Electrical Engineering, Ming Chi University of Technology, New Taipei City 24300, Taiwan; 5 Department of Electronic Engineering, Chung Yuan Christian University, Taoyuan City 32023, Taiwan; chrischen@cycu.edu.tw (S.-L.C.); m11252012@mail.ntust.edu.tw (C.-H.C.); 6 Program in Electrical Engineering & Computer Science, Chung Yuan Christian University, Taoyuan City 32023, Taiwan; m16121158@gs.ncku.edu.tw (Y.-J.H.); a123456749@kimo.com (J.-K.C.); 7 Department of Electronic Engineering, National Cheng Kung University, Tainan City 70101, Taiwan; 8 Ateneo Laboratory for Intelligent Visual Environments, Department of Information Systems and Computer Science, Ateneo de Manila University, Quezon City 1108, Philippines; pabu@ateneo.edu

**Keywords:** panoramic radiographs, image enhancement, tooth localization, image processing, convolutional neural network, clinical decision support systems

## Abstract

**Background/Objectives:** Panoramic radiography (PANO) is widely utilized for routine dental examinations, as a single PANO image captures most anatomical structures and clinical findings, enabling an initial assessment of overall dental health. Dentists rely on PANO images to enhance clinical diagnosis and inform treatment planning. With the advancement of artificial intelligence (AI), the integration of clinical data and AI-driven analysis presents significant potential for supporting medical applications. **Methods:** The proposed method focuses on the segmentation and localization of impacted third molars in PANO images, incorporating Sobel edge detection and enhancement methods to improve feature extraction. A convolutional neural network (CNN) was subsequently trained to develop an automated impacted tooth detection system. **Results:** Experimental results demonstrated that the trained CNN achieved an accuracy of 84.48% without image preprocessing and enhancement. Following the application of the proposed preprocessing and enhancement methods, the detection accuracy improved significantly to 98.66%. This substantial increase confirmed the effectiveness of the image preprocessing and enhancement strategies proposed in this study. Compared to existing methods, which achieve approximately 90% accuracy, the proposed approach represents a notable improvement. Furthermore, the entire process, from inputting a raw PANO image to completing the detection, takes only 4.4 s. **Conclusions:** This system serves as a clinical decision support system for dentists and medical professionals, allowing them to focus more effectively on patient care and treatment planning.

## 1. Introduction

An impacted tooth is defined as one that fails to fully erupt into its correct occlusal position [1]. The primary causes of tooth impaction include insufficient space within the dental arch, ectopic eruption, genetic factors, and physical obstruction by adjacent teeth or bone structures [2]. Among these, insufficient arch space and ectopic eruption are the most prevalent causes, frequently observed in mandibular third molars [3].

Between 50% and 80% of the global population are estimated to have at least one impacted tooth during their lifetime [4]. Impacted third molars are commonly classified using the Pell and Gregory classification system, which evaluates two anatomical relationships: First, impaction depth is defined as Class A, B, or C depending on the vertical relationship between the crown of the impacted tooth and the height of the adjacent second molar. Second, the available space adjacent to the second molar is evaluated to determine whether it is sufficient to accommodate the crown of the impacted tooth, leading to a classification of Class I, II, or III [5]. Impacted teeth are associated with an increased risk of complications, including cyst formation, lesions, root resorption, pericoronitis, periodontitis, and tumor development, all of which can adversely affect adjacent teeth [6,7]. Therefore, routine oral examinations are critical for the early detection and management of impacted teeth, helping to mitigate potential complications and preserve oral health.

In recent years, deep learning has been extensively applied across various domains [8,9]. Incorporating AI into medical practice has led to more efficient diagnostic procedures and improved clinical outcomes [10]. For instance, a machine learning-based application for assessing dental erosion has been developed [11], which can operate on a smartphone platform, making dental evaluation more accessible and convenient. In addition, several AI-based systems have been developed, incorporating convolutional neural networks (CNNs) to detect periapical lesions [12]. Specifically, faster region-based convolutional neural networks (Faster R-CNN) have been proposed for automated tooth detection and numbering [13], providing a comprehensive framework that integrates lesion detection with dental numbering for PANO image analysis.

Building on these developments, PANO is a widely utilized X-ray imaging technique for diagnosing impacted teeth [14]. It is the most commonly employed modality for evaluating overall oral health, offering the advantage of capturing a broad anatomical area with minimal radiation exposure [15]. Recent studies have also showed the feasibility of applying deep learning for the accurate detection of impacted teeth in PANO images. The You Only Look Once (YOLO) model has been utilized for impacted tooth detection, highlighting the potential of object detection techniques in dental imaging [16]. Additionally, multiple CNN architectures have been explored, with the InceptionResNetV2 model achieving an accuracy of 92% in impacted tooth identification [17]. Although both approaches reported an identification accuracy exceeding 90%, the image enhancement techniques employed were relatively simplistic or, in some cases, absent altogether. This highlights the opportunity for further improvement through the adoption of more advanced image preprocessing methods.

To further enhance model performance, various image enhancement techniques have been introduced. One approach utilizes Gaussian high-pass filtering to isolate and eliminate non-target noise, thereby improving the classification and localization of individual teeth [18]. Building on the concept of enhancing critical features prior to model training, Sobel edge detection has also been proposed as a preprocessing step [19], significantly improving the visibility of key anatomical structures in dental images. Moreover, enhanced datasets have been employed to train CNN models with a focus on emphasizing symptomatic features [20]. Experimental results indicate that image enhancement techniques can lead to a 6% increase in detection accuracy, confirming the positive impact of preprocessing on CNN model performance. Collectively, these studies underscore the crucial role of image enhancement in dental diagnostics. By improving the visibility of symptomatic features, advanced preprocessing methods substantially contribute to the accuracy and robustness of deep learning-based diagnostic systems. Consequently, integrating feature enhancement techniques into the training pipeline has emerged as an effective strategy for optimizing CNN-based detection and classification in medical imaging.

In this study, an automated system was proposed for the detection of impacted teeth in PANO images through a multi-stage framework that included double-tooth cropping, image enhancement, and deep learning-based classification. A segmentation method was first applied to isolate individual double teeth from the PANO images, thereby reducing the interference caused by overlapping or adjacent teeth during CNN-based recognition. Following segmentation, image enhancement methods were employed to emphasize symptomatic features critical for accurate diagnosis. The enhanced images were then analyzed by using a CNN model to detect the presence of impacted teeth. Experimental results showed that the integration of image preprocessing, segmentation, enhancement, and CNN training significantly improved detection performance, achieving a maximum accuracy of 98.66%. The system outputted visual identification results directly on the PANO images, offering a practical diagnostic decision support tool for dentists and medical professionals. By streamlining the detection process and enhancing diagnostic precision, this system has the potential to facilitate more efficient and informed follow-up treatments for patients.

## 2. Methods

In this study, a CNN model was employed to automatically identify diseased teeth from PANO images captured using dental X-ray imaging systems. To ensure clinical relevance, all PANO images used in this study were obtained from the image database of Chang Gung Memorial Hospital in Taoyuan, Taiwan. The images were randomly selected by a dentist after all personally identifiable information had been removed. The study protocol was reviewed and approved by the Institutional Review Board (IRB) of Chang Gung Memorial Hospital, with the approval number 202002030B0C504. All image acquisition and annotation of the patients’ oral conditions were conducted by an attending dentist with over three years of clinical experience.

**Figure 1:**
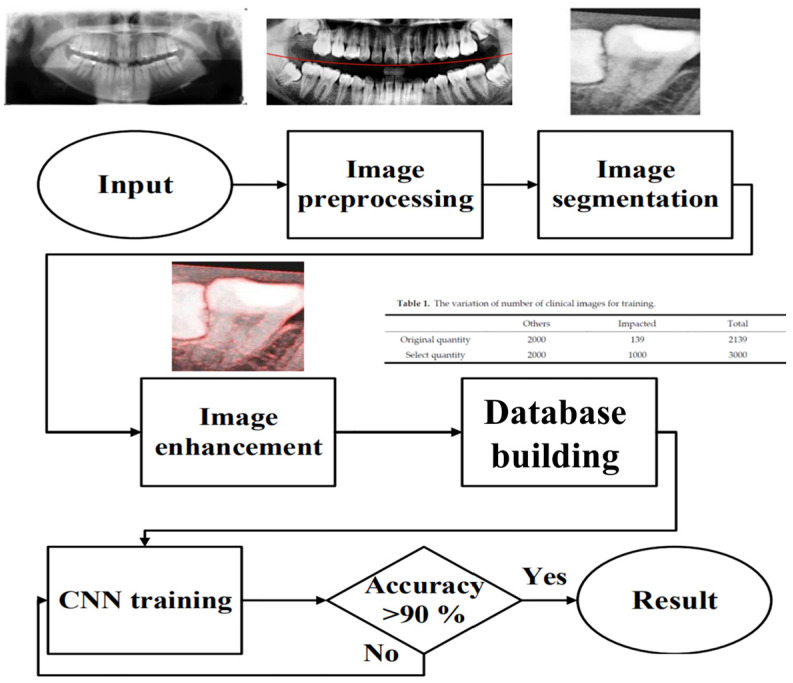
A flow diagram of this study.

To effectively reduce diagnostic time and improve clinical efficiency, this study proposed a fully automated system for the identification of impacted teeth, in which all processing stages were executed without manual intervention. The overall workflow of the proposed system is shown in Figure 1. These steps included image preprocessing, cropping and positioning in PANO images, symptom enhancement algorithms, and the establishment of a CNN training database. Through image preprocessing and segmentation, the input PANO images were standardized and cropped to a size of 200×300, each containing two teeth. The standardized double-teeth images were then enhanced to improve the visibility of the symptoms. The enhanced images were used to train the CNN model, which was capable of recognizing impacted teeth.

### 2.1. Image Preprocessing

**Figure 2:**
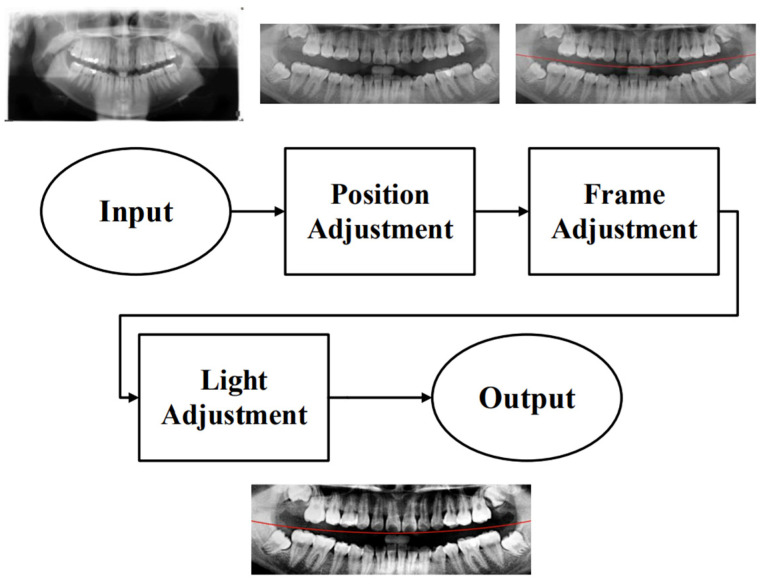
A flow diagram of the preprocessing steps in this study. The red line indicates the boundary between the upper and lower jaws (maxilla and mandible) in this study.

Due to the excessive amount of information present in unprocessed PANO images, irrelevant features may negatively impact the training and recognition performance of the CNN model. To construct an optimal dataset for this study, three preprocessing steps were applied: position adjustment, frame adjustment, and light adjustment, as shown in Figure 2.

#### 2.1.1. Position Adjustment

**Figure 3:**
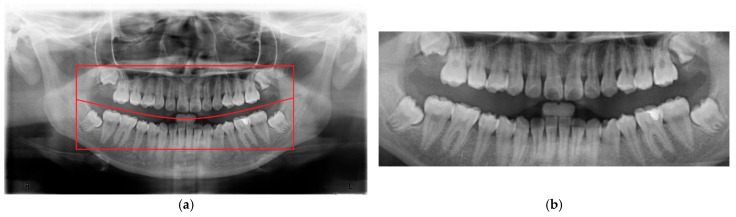
An example of finding the initial average block. (**a**) The original PANO image with the red line representing the ideal cropping line. (**b**) The result of position adjustment.

To minimize the influence of non-target regions on the recognition process, position adjustment was implemented as a critical preprocessing step. Given the variability in oral cavity positioning across different patients, the average location of the oral cavity was estimated using 100 randomly selected PANO images. Based on this analysis, the target region was determined to be approximately 625×1650 pixels, as shown in Figure 3. Using this reference region, only the relevant portions of each original PANO image were retained. This cropping process effectively eliminated extraneous areas outside the alveolar bone, thereby enhancing the accuracy and efficiency of the subsequent recognition tasks performed by the CNN model.

#### 2.1.2. Frame Adjustment

**Figure 4:**
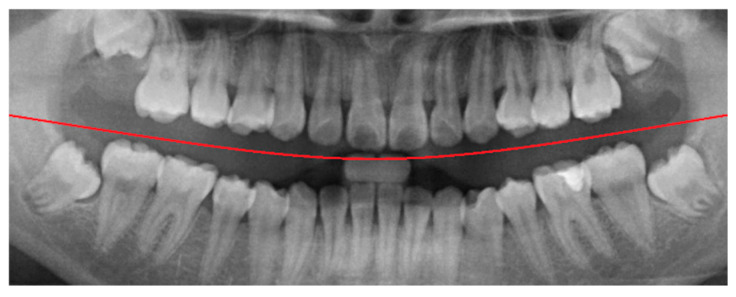
The result of the curve and central point are labeled. The red line indicates the boundary between the upper and lower jaws (maxilla and mandible) in this study.

In the framing adjustment step, the central point of the oral structure was first identified. This point corresponded to the vertex of a second-order polynomial curve. The gap between the upper and lower jaws was then modeled using a quadratic curve that passes through the central point and connects to two boundary points on the image frame [21], as described by Equation (1). The resulting second-order curve used to represent the jaw separation is shown in Figure 4.

(1) $$y\left(x\right)=ax^{2}+bx+c$$

#### 2.1.3. Light Adjustment

**Figure 5:**
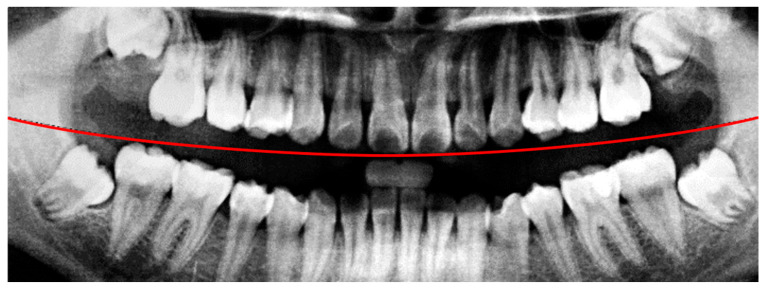
The result of image preprocessing through light adjustment.

Insufficient brightness in the original PANO images may lead to inaccurate segmentation and hinder the extraction of relevant features. To mitigate this issue, background illumination was estimated and corrected using an opening operation from mathematical morphology [22], as defined in Equation (2). In this formulation, $A\left(x,y\right)$ represents the original image and $S\left(i,j\right)$ denotes a disk-shaped structuring element used in the morphological operation. The result of this illumination correction process is shown in Figure 5.

(2) $$A\circ S=\left(A\left(x,y\right)-S\left(i,j\right)+S\left(i,j\right)\right)$$

### 2.2. Image Segmentation

**Figure 6:**
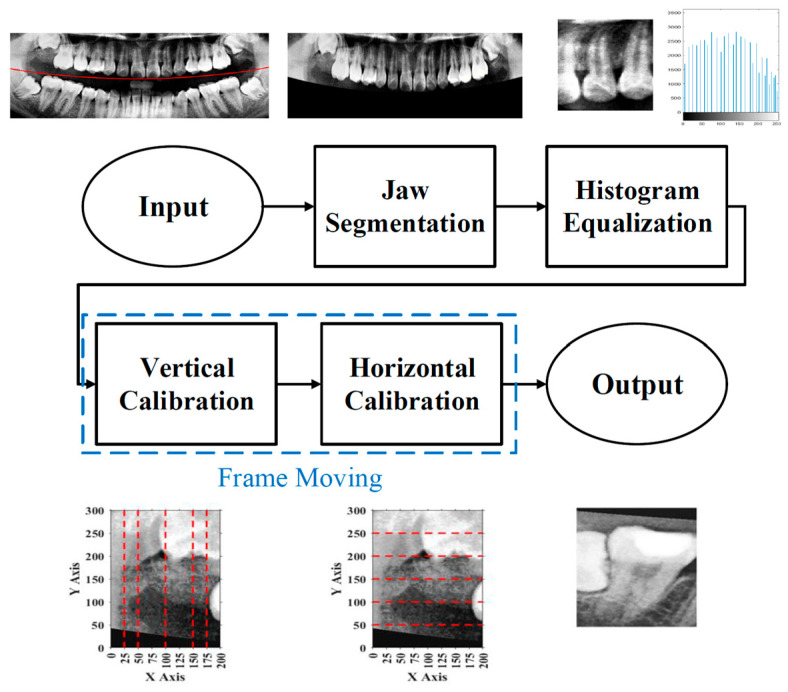
The flow diagram illustrates the segmentation steps.

The primary objective of the image segmentation step was to identify the upper and lower jaws and to isolate each jaw into individual tooth images. This process enabled more precise analysis and classification at the double-tooth image. Figure 6 presents a flow diagram of the image segmentation procedure, showing the complete process from the original PANO image to the generation of the double-tooth images.

#### 2.2.1. Jaws Segmentation

**Figure 7:**
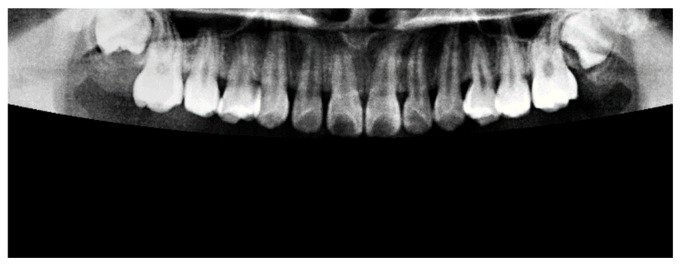
The result of masking for jaw segmentation.

Following the preprocessing step, it was necessary to isolate the target jaw by masking the opposing jaw. This was achieved by applying a binary mask based on a defined condition [22], as described in Equation (3). The masking operation was performed either above or below the second-order curve used to approximate the gap between the jaws. In this context, $A_{\left(i,\;j\right)}$ represents the original pixel value, while $B_{\left(i,j\right)}$ denotes the modified pixel value after applying the mask. An example of the masking result is shown in Figure 7, where the upper jaw was successfully extracted, and the lower jaw was fully masked out, effectively eliminating irrelevant anatomical structures from the image.

(3) $$B_{\left(i,j\right)}=\left\{\begin{array}{c} \begin{array}{cc} 0, & \;\;\;if\;A_{\left(i,\;j\right)}<128 \end{array}\\ \begin{array}{cc} A_{\left(i,\;j\right)}, & \;\;\;otherwise \end{array} \end{array}\right.$$

#### 2.2.2. Histogram Equalization

Due to the occasional indistinct appearance of bone structures in PANO images, slight variations may occur during the judgment of anatomical boundaries. To enhance precision during frame alignment and segmentation, histogram equalization was applied to improve image contrast. This method increases the global contrast of an image, thereby enhancing the visibility of subtle features [23].

**Figure 8:**
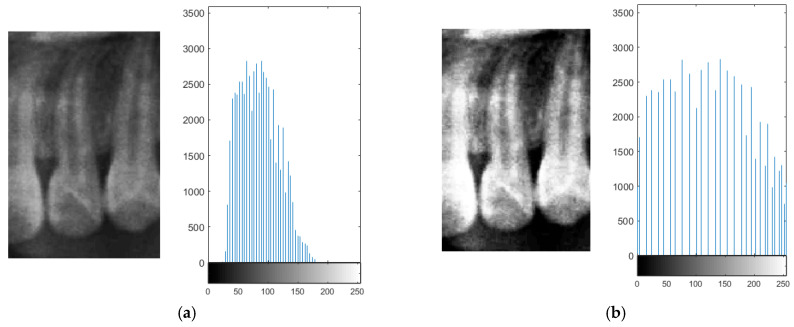
The compared result and histogram of the histogram equalization. (**a**) The original teeth. (**b**) The adjusted teeth.

In the segmentation step, both the teeth and non-object regions such as the background and interproximal spaces between the teeth exhibited low local contrast, which could lead to inaccuracies when isolating teeth. By applying histogram equalization, bone structures and dental boundaries became more pronounced, contributing to improved segmentation accuracy. The visual improvement resulting from histogram equalization is shown in Figure 8.

#### 2.2.3. Frame Moving

**Figure 9:**
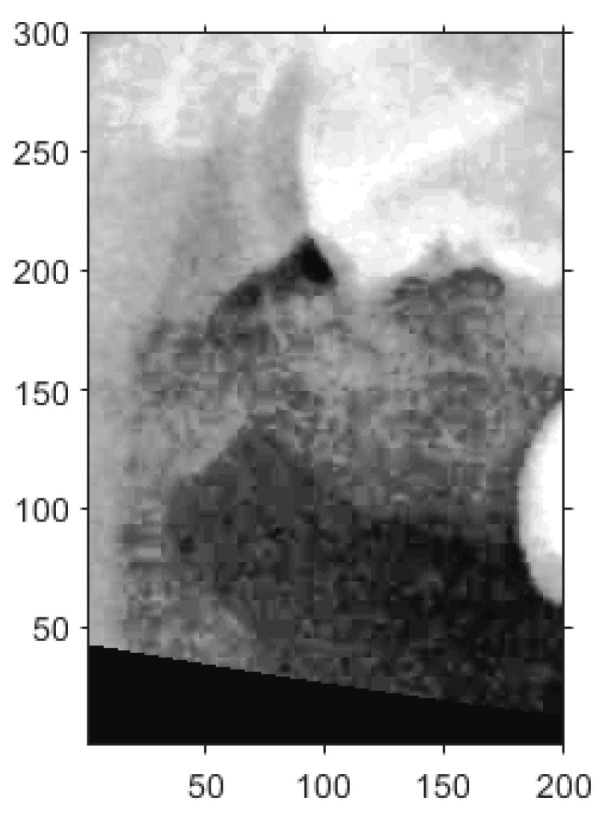
An example of a 200×300 image cropped from a PANO image.

Two common segmentation approaches exist, with one involving the masking of the target tooth to exclude non-relevant areas, while the other segments the teeth based on their individual contours [24,25]. However, because the identification of impacted teeth requires the contextual presence of at least two adjacent teeth, both segmentation methods were modified accordingly in this study. Through empirical evaluation and implementation, a frame size of 200×300 pixels was determined to be the most effective for capturing a sufficient diagnostic region, as shown in Figure 9.

The upper-left corner coordinates of each segmentation frame were recorded to assist in subsequent double-tooth localization. A Cartesian coordinate system was established within each frame to standardize the positioning. Since the pixel intensities of the teeth and bone structures were generally higher than those of the surrounding soft tissue due to differences in density, the frame was adjusted accordingly to ensure proper coverage of the target tooth through both vertical and horizontal alignment.

**Figure 10:**
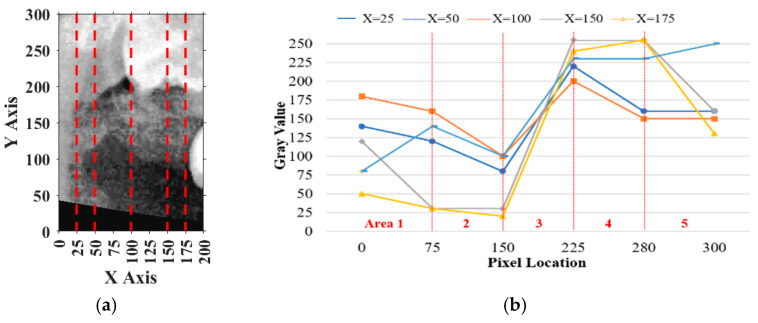
An example of a 200×300 image cropped from a PANO image in vertical adjustment. (**a**) An example for the upper jaw (The red dashed line marks the vertical curve in pixel space). (**b**) The vertical curve of the gray pixel at x = 25, 50, 100, 150, and 175.

For vertical adjustment, the system first verified whether the crown of the target tooth was included within the segmentation frame. To achieve this, five columns of pixel intensity values, each 300 pixels in height, were extracted from the image at fixed x-coordinates: x = 25, 50, 100, 150, and 175. The resulting intensity distributions were used to guide the alignment and are shown in Figure 10a.

Pixels on the *y*-axis ranging from 0 to 75 were defined as Area 1, from 75 to 150 as Area 2, from 150 to 225 as Area 3, from 225 to 280 as Area 4, and from 280 to 300 as Area 5. The sum of Area 1 and Area 5 was defined as P1, the sum of Areas 1, Areas 2, and Areas 5 was defined as P2, and the remaining pixels were summed to obtain P3. The result should perfectly satisfy P3>P2 and the percentage of P1<50%, which meant the vertical place of the tooth was included correctly. If more than half of the curves in Figure 10b satisfied these conditions, the vertical position was verified. Otherwise, the frame was recalibrated by shifting it downward in 10-pixel increments until the criteria were met.

**Figure 11:**
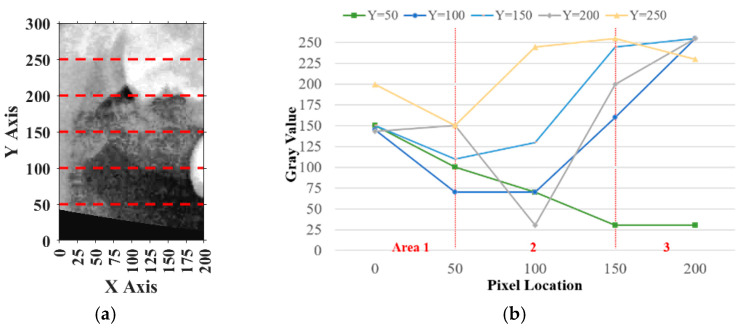
An example of a 200×300 image cropped from a PANO image in horizontal adjustment. (**a**) An example for the upper jaw (The red dashed line marks the horizontal curve in pixel space.). (**b**) The horizontal curve of the gray value at y = 50, 100, 150, 200, and 250.

Similarly, for horizontal adjustment, the method followed the same principle as vertical calibration. Five horizontal lines of pixel intensity values were extracted at fixed y-coordinates: y = 50, 100, 150, 200, and 250, as shown in Figure 11a.

For each horizontal line, the pixels on the *y*-axis ranging from 0 to 50 in the plot were defined as Area 1, pixels from 50 to 150 were defined as Area 2, and pixels from 150 to 200 were defined as Area 3. The sum of Area 1 and Area 3 was P4, while the remaining area values were summed to obtain P5. The result should have satisfied P4>P5, and the deviation between Area 1 and Area 3 must have been smaller than 20%, indicating that the horizontal plane of the tooth was correctly included. If more than half of the horizontal lines met these requirements, the horizontal location was verified, as shown in Figure 11b. Otherwise, the frame needed to be recalibrated. During calibration, the frame was moved 5 pixels from left to right each time until the specifications were met. The movement range was limited to 125 pixels because the average tooth width was approximately 175 to 200 pixels.

### 2.3. Image Enhancement

The objective of the enhancement process was to emphasize the contours of the teeth, thereby facilitating the construction of an effective CNN model and improving the classification accuracy. To this end, both Sobel edge detection and Canny edge detection were evaluated as part of the enhancement process.

Sobel edge detection is capable of computing gradients along specific axes independently, as defined in Equations (4) and (5). Here, $f_{x}^{\prime }\left(x,\;y\right)$ and $f_{y}^{\prime }\left(x,\;y\right)$ represent the horizontal and vertical gradients, respectively. These directional gradients can be combined to form a two-dimensional gradient magnitude, constituting the Sobel operator used for edge detection.

(4) $$\begin{array}{c} f_{x}^{\prime }\left(x,\;y\right)=f\left(x-1,\;y+1\right)+2f\left(x,\;y+1\right)\\ +f\left(x+1,\;y+1\right)-f\left(x-1,\;y-1\right)\\ -2f\left(x,\;y-1\right)-f\left(x+1,\;y-1\right) \end{array}$$

(5) $$\begin{array}{c} f_{y}^{\prime }\left(x,\;y\right)=f\left(x-1,\;y-1\right)+2f\left(x-1,\;y\right)\\ +f\left(x-1,\;y+1\right)-f\left(x+1,\;y-1\right)\\ -2f\left(x+1,\;y\right)-f\left(x+1,\;y+1\right) \end{array}$$

(6) $$G\;\left[f\left(x,\;y\right)\right]=\left|f^{\prime }x\left(x,\;y\right)\right|+\left|f^{\prime }y\left(x,\;y\right)\right|$$

**Figure 12:**
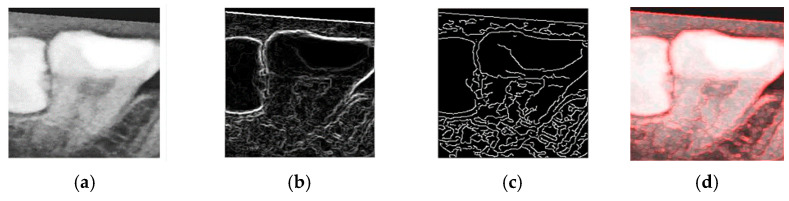
The results of image enhancement on a tooth image. (**a**) Original image. (**b**) The result of Sobel edge detection. (**c**) The result of Canny edge detection. (**d**) The final enhancement results.

To determine the most effective enhancement method, this study compared the visual outcomes of Sobel and Canny edge detection, as shown in Figure 12a–c. Based on visual inspection, the images processed with Canny edge detection appeared blurrier and contained more noise compared to those enhanced using the Sobel method. Consequently, Sobel edge detection was selected as the preferred enhancement method. To further highlight the image features, this study applied a color-overlay approach by superimposing the Sobel edge map onto the original image, thereby enhancing structural visibility. The resulting enhanced image is shown in Figure 12d.

### 2.4. Database Building

Due to the imbalance between the two classification categories, “Impacted” and “Others”, the training dataset was subject to a class imbalance issue. Specifically, the “Others” category contained approximately 2000 single-tooth images, whereas the “Impacted” category included only 139 images. To address this imbalance, data augmentation methods were applied to expand the “Impacted” dataset. Using image rotation and horizontal flipping, the number of “Impacted” samples was increased to 1000. To balance the dataset, a subset of 1000 samples from the “Others” category was randomly selected by the system. An overview of the clinical dataset used in this study is provided in Table 1.

### 2.5. Deep Learning

Deep learning enables both supervised and automated feature learning through hierarchical feature extraction. It mimics the way the human brain processes information by passing learned representations through multiple layers. This layer-by-layer learning process allows the model to develop increasingly complex and discriminative decision-making capabilities. In this study, five deep learning architectures, AlexNet, VGG19, GoogLeNet, SqueezeNet, and Xception, were employed as disease recognition models. These models are composed of convolutional layers, fully connected layers, and pooling layers, which together enable highly effective performance in image classification tasks.

To further enhance the training efficiency, this study adopted a transfer learning approach. Through the fine-tuning of previously trained networks, this method supported the application of learned knowledge to different domains with shared feature spaces. Compared to training a model from scratch, this method was more flexible and less computationally intensive, as it eliminated the need to rebuild the entire network manually. In this process, a pre-trained network was selected and modified to suit the specific problem domain, followed by fine-tuning with domain-specific data. Since data across different domains often share common features, transfer learning enables previously trained models to accelerate the training of new models while maintaining high performance.

In conjunction with transfer learning, tuning the model hyperparameters was critical for identifying the optimal CNN configuration [18]. It is important to distinguish between parameters and hyperparameters: parameters are learned by the model during training, while hyperparameters are predefined settings that guide the training process. Different combinations of hyperparameters effectively define different models. In this study, three key hyperparameters, Learning Rate, Max Epoch, and Mini BatchSize, were systematically adjusted to optimize the model performance.

The software and hardware environment used in the study are shown in Table 2. In terms of transfer learning, Matlab was used for software development. For CNN model training and accelerated deep learning, this study used a Nvidia Geforce RTX 3060 GPU (NVIDIA Corporation, Santa Clara, CA, USA) in terms of the hardware performance.

## 3. Results

This section presents the performance results of segmentation, enhancement, and the CNN. The trained CNN model was finally used to preliminarily judge the disease of the teeth, by letting the model predict whether the patient had diseased teeth or not. After that, the model reconciled the results and output them as a table.

To assess the model accuracy, the validation set was used as input for the trained network during testing. Evaluation indicators play a crucial role in assessing the performance of CNN models. The confusion matrix is a widely used evaluation method and is particularly effective for binary classification models. It provides a visual representation of the model’s correct and incorrect predictions. In the matrix, True Positives (Tp) and True Negatives (Tn) indicate cases where the model’s predictions match the actual outcomes, while False Positives (Fp) and False Negatives (Fn) represent incorrect predictions. An example of the confusion matrix is shown in the Table 3.

To comprehensively evaluate the performance of the proposed model, four key metrics were employed: Accuracy, Precision, Recall, and F1 score. Accuracy offered an overall assessment of the model’s correctness across all classes. Precision reflected the model’s ability to minimize false positives, while recall indicated how effectively the model captured relevant instances and avoided false negatives. The F1 score provided a balanced metric that accounted for both types of classification errors, false positives and false negatives, as shown in Equations (7)–(9).

(7) $$Accuracy=\frac{Tp+Tn}{Tp+Fp+Tn+Fn}$$

(8) $$Precision=\frac{Tp}{Tp+Fp}$$

(9) $$Recall=\frac{Tp}{Tp+Fn}$$

(10) $$F1\;Score=2\times \frac{\left(Precision\times Recall\right)}{Precision+Recall}$$

**Figure 13:**
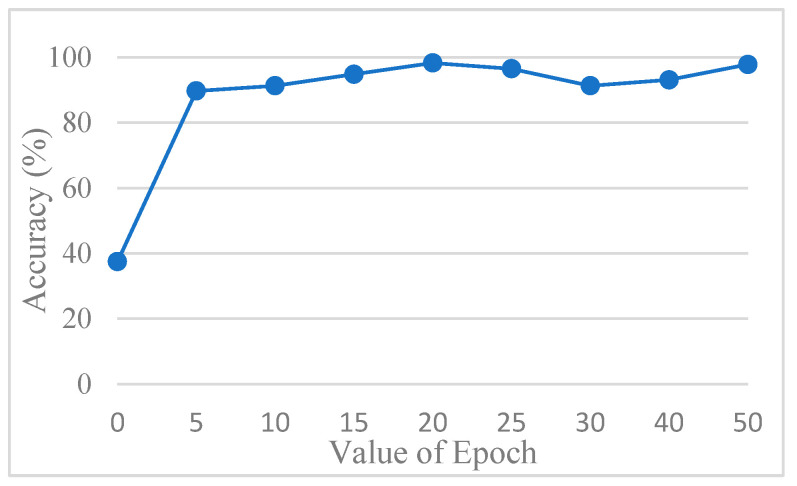
The accuracy of AlexNet in different epochs.

**Figure 14:**
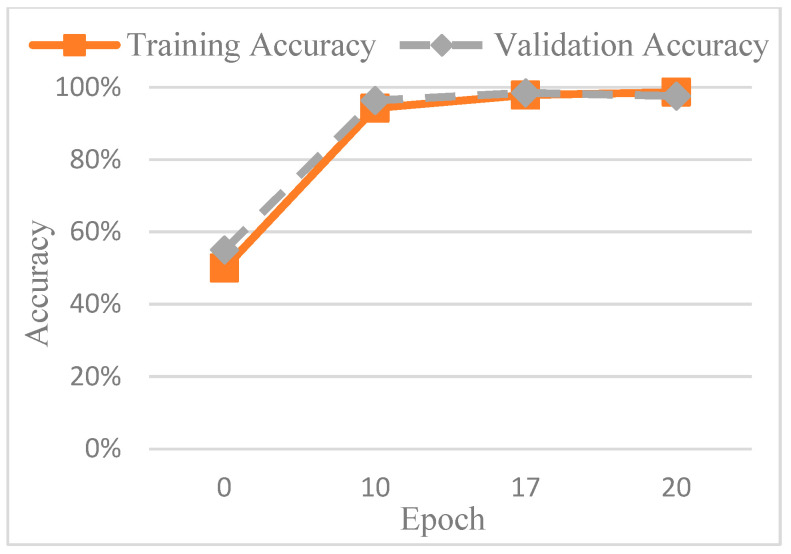
The loss function performance of AlexNet.

**Figure 15:**
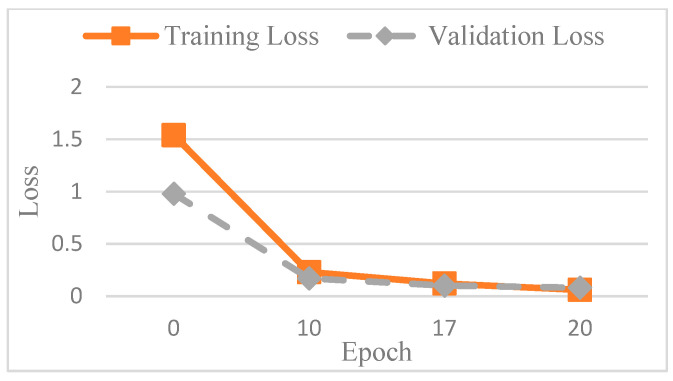
The loss of AlexNet in different epochs.

The accuracy of the CNN model was determined by comparing its predictions with the ground truth annotations of the images. To avoid overfitting, this study used AlexNet for testing. In order to find out the best value for the epoch, it was tested several times. Figure 13 shows the accuracy trend across the epochs, and based on these observations, the number of epochs was set to 20 to balance training performance and overfitting risk. The final training accuracy curve of AlexNet is shown in Figure 14, while the corresponding loss function performance is presented in Figure 15.

**Figure 16:**
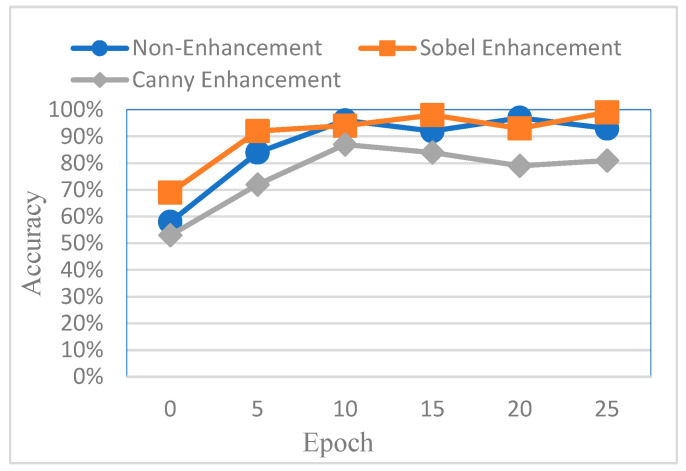
Comparison of the accuracy of the training process for the original image, Sobel enhancement, and Canny enhancement.

To evaluate the effectiveness of different image enhancement methods for impacted tooth detection, this study conducted a comparative analysis using AlexNet as the baseline model. The classification accuracy was compared across three input types: original images, images enhanced using Canny edge detection, and images enhanced using Sobel edge detection. The results are shown in Figure 16. It was observed that images processed with Canny edge detection yielded the lowest training performance, primarily due to the additional noise introduced by the Canny algorithm, which negatively affected feature clarity and model learning.

However, despite the improved accuracy achieved through Sobel edge enhancement, the overall performance remained suboptimal. To further enhance CNN effectiveness, this study explored a color transformation method to enhance CNN performance by extracting more information and features from the image. The experimental results showed a significant improvement, as summarized in Table 4. For identical image inputs, the application of the color-based enhancement method yielded a 16.79% increase in classification accuracy compared to the non-enhanced version. This confirmed that the proposed enhancement approach effectively improved CNN training efficiency and model performance. The confusion matrix corresponding to the final model results is presented in Table 5.

In addition to evaluating the training time and execution performance of the various models, this study also compared the results with more recent research, as shown in Table 6. This included a 2022 study that utilized Inception V3 [26], a 2025 study that integrated YOLO (You Only Look Once) and RT-DETR (Real-Time Detection Transformer) [16], and another 2025 study that employed Xception [17]. The results of this work was impressive. Compared to the other methods, this study achieved the highest accuracy, precision, and F1. Only the value for recall was slightly lower than that of the method from [16]. Our results showed that the model’s accuracy and reliability can be improved by adjusting the image enhancement method or incorporating multiple models for evaluation.

**Figure 17:**
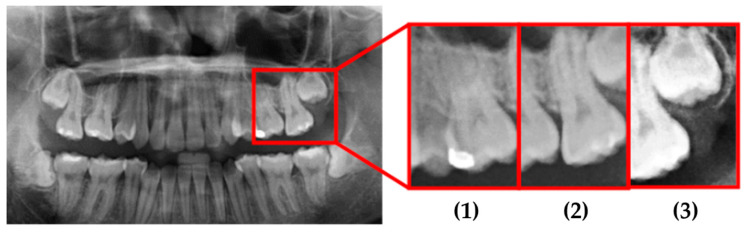
Example data that was cropped by this study.

This study used actual clinical images, for example, as shown in Figure 17 and Table 7. When the original PANO image was input, it underwent image processing and cropping for calibration. Subsequently, image enhancement was applied to emphasize the symptom features. Finally, the enhanced image was fed into the CNN for classification. From the input of the original PANO image to the completion of identification, the process only took 4.4 s. Specifically, in this system, cropping the original PANO image into double-teeth images took 2.6 s, while image enhancement and CNN identification of impacted teeth took only 1.8 s. Image diagnosis through the final model approached the best results. Impaction was found to be correctly and quickly diagnosed with a high degree of reliability for each tooth.

## 4. Discussion

For dentists, it is essential to quickly diagnose and treat diseased teeth. This study tested five common types of CNN, namely AlexNet, GoogLeNet, VGG-19, SqueezeNet, and Xception. The model performance and results are detailed in Table 8. From the results, it was found that both GoogLeNet and AlexNet achieved an accuracy of 98%, with the accuracy of AlexNet being only 0.23% lower than GoogLeNet. However, in terms of training time, AlexNet took 3 min and 18 s, significantly shorter than GoogLeNet. Although the results showed that SqueezeNet took only 19 s to train, its accuracy was only 88.10%. In terms of the execution time, SqueezeNet and Xception required only 13 milliseconds, while AlexNet took 33 milliseconds. Therefore, considering both the accuracy and execution speed, this study selected AlexNet as the CNN model.

Although this study used an older model for training, the proposed preprocessing, image segmentation, and enhancement methods effectively improved the model’s training efficiency. This was confirmed by Table 6 and Table 8, where the accuracy of AlexNet, GoogLeNet, VGG19, and Xception all exceeded that of the model proposed in method [17]. The most notable highlight was that both AlexNet and GoogLeNet achieved an accuracy of 98%. In the future, this study will explore the use of more advanced models combined with the proposed image processing methods to achieve even higher accuracy.

The main goal of this study was to improve diagnostic efficiency by automatically detecting impacted teeth from PANO images to reduce the burden on dentists during detection. This study aimed to enhance diagnostic efficiency by automating the detection of impacted teeth in PANO images. The proposed method involved automatic segmentation, image enhancement, and the training of a CNN for object recognition. The novelties of the proposed method are as follows:

1. An advanced method for PANO image preprocessing.

Using the disc structuring element and the opening operation in mathematical morphology, the background of brightness adjustment was found so that the light could be balanced in the PANO image. This helps to initialize frame segmentation to reduce the influence of non-target objects in the judgment process.

2. A new method for segmentation and localization in PANO images.

The second-order curve can not only separate the upper and lower jaws, but also completely remove the non-target teeth under masking conditions. The influence of image segmentation on edge detection was improved to some extent, and crown retention ensured better single-tooth images in the enhanced parts. This method was reinforced to minimize unnecessary areas for target teeth and improve training efficiency.

3. Image enhancement methods for impacted teeth.

We selected the Sobel edge detection method to reduce blur noise. Since this article focused on impacted teeth, the image of the entire tooth, including the crown and root, was primarily strengthened. Combined with edge enhancement, the disease detection accuracy was improved from 84.48% to 98.66%.

4. A more accurate impacted teeth detection system.

From these results, there were four models with an accuracy rate above 90%. Among them, the highest accuracy model was AlexNet, which reached 98.66%. Compared with the other methods, this accuracy rate was improved by almost about 9.62%.

## 5. Conclusions

This study proposed an automated system for identifying impacted teeth in PANO images, achieving a final accuracy of up to 98.66%. The results clearly demonstrated that the proposed method contributes to advancements in PANO image preprocessing, segmentation, localization, feature enhancement for impacted teeth, and detection accuracy. These contributions have the potential to improve the effectiveness and efficiency of dental image analysis for diagnosing and managing impacted teeth. In addition, the proposed system offers a practical clinical decision support system for dentists and medical professionals. After a patient undergoes panoramic radiography, the image can be directly input into the system, which automatically identifies and highlights the impacted tooth regions on the original PANO image. This not only provides dentists with immediate diagnostic insights but also significantly reduces the time and manpower required for manual annotation and interpretation.

Nevertheless, certain limitations remain. These include limited adaptability to various radiographic formats, a focus on impacted teeth rather than broader dental conditions, and the trade-off between model accuracy and computational complexity. Future work will aim to incorporate the detection of additional dental conditions to build a more comprehensive model. Furthermore, the integration of advanced architecture such as Faster R-CNN is planned to address diverse clinical needs and enable high-efficiency, rapid diagnostic support in dental practice.

## Figures and Tables

**Table 1:** The variation in the number of clinical images used for training.

	Others	Impacted	Total
Original quantity	2000	139	2139
Select quantity	2000	1000	3000

**Table 2:** The hardware and software platforms.

Hardware Platform	Version
CPU	Intel Core i7-11370H
GPU	Geforce GTX 3060
DRAM	DDR4 2666-24G
**Software Platform**	**Version**
MATLAB	R2021a
Deep Network designer	14.2

**Table 3:** An example of the confusion matrix.

	True	Positive	Negative
Predicted			
Positive		Tp	Fp
Negative		Fn	Tn

**Table 4:** The comparison between enhancement methods and the original image for CNN identification results.

	Original Images	Sobel Edge Detection
Validation Accuracy	84.48%	98.66%
Validation Loss	0.42	0.11
Image	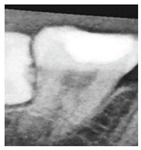	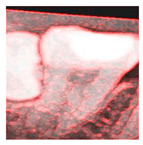

**Table 5:** The confusion matrix for the AlexNet model.

	Impacted	Others
Impacted (P)	819 (TP) 49.94%	21 (FN) 1.28%
Others (N)	1 (FP) 0.06%	799 (TN) 48.72%

**Table 6:** The comparison between different proposed methods.

	Method in [26]	Method in [16]	Method in [17]	This Study
Accuracy	92.59%	97.5%	90%	98.66%
Precision	93.55%	89.1%	N/A	99.80%
Recall	93.55%	98.4%	N/A	97.50%
F1	93.55%	93.5%	N/A	99.21%

N/A: not applicable.

**Table 7:** The identified results by this study.

Tooth Position	Origin Data	This Work
(1)	Normal	98.35% to be Normal
(2)	Normal	99.23% to be Normal
(3)	Impacted	99.87% to be Impacted

**Table 8:** The comparison between different model performances.

	AlexNet	GoogLeNet	VGG19	SqueezeNet	Xception
Accuracy	98.66%	98.89%	95.20%	88.10%	95.10%
Training Time (second)	198	1427	1920	19	539
Elapsed Time (millisecond)	33	38	105	13	13.58

## Data Availability

The data presented in this study are available on request from the corresponding author.
